# Interpretive review: Semiochemicals in domestic pigs and dogs

**DOI:** 10.3389/fvets.2022.967980

**Published:** 2022-10-25

**Authors:** John J. McGlone, Courtney Archer, Madelyn Henderson

**Affiliations:** Laboratory of Animal Behavior, Physiology and Welfare, Texas Tech University, Lubbock, TX, United States

**Keywords:** dog, semiochemical, pheromone, interomone, olfaction, pig

## Abstract

This interpretive review includes discussion of the available scientific literature with interpretations by the authors. The broad field of semiochemicals can be confusing to scientists and consumers. This review attempts to summarize the known scientific studies for pig and dog semiochemicals while at the same time attempting to refine our use of terminology. The specific objectives of this interpretive review are to summarize and interpret much of the key scientific literature (but not the lay literature) on semiochemicals in pigs and dogs to include (1) definitions of semiochemicals and related molecules including pheromones, (2) to briefly summarize olfactory organs, and (3) and to examine the scientific literature for semiochemical mechanisms and applications in dogs and pigs (two domesticated species with known olfactory acuity). Dogs and pigs have olfactory features that are similar in that they both lack certain olfactory organs (Grueneberg ganglion and Septal Organ) and they have a small vomeronasal organ (VNO) without some major receptors that are found in other species. The primary olfactory organs for both pigs and dogs are the main olfactory epithelium and perhaps the trigeminal nerve. Several examples of pheromones activating the brain *via* the MOE or Trigeminal nerve rather than the VNO challenge the concept that the VNO is the site of pheromone sensing. We believe it is not appropriate to label something a pheromone when evidence is not available to show that it is a pheromone. We offer definitions for the terms semiochemicals, pheromones, interomones and others and then determine if the evidence is sufficient to call certain semiochemicals a pheromone. Here we review mixed, largely negative, scientific reports of the efficacy of some products labeled as “pheromones” that are more appropriately called semiochemicals. Interomones can have a more powerful effect on dog behavior and physiology than semiochemicals marketed as pheromones. Because marketing of semiochemicals is far ahead of the science, bringing some logic and uniformity to the field will benefit animals and hopefully cause less consumer confusion. Semiochemicals have the potential to offer powerful solutions to behavioral problems using more naturally occurring molecules.

## Introduction

All farm and companion mammals are macrosmatic species. This distinction means that the olfactory bulbs and/or other chemical sensory organs are large in size compared with microsmatic species such as many primates (1). We consider species with large numbers of olfactory receptors to have greater olfactory acuity than species with fewer functional olfactory receptors such as birds; however, the number of olfactory receptors does not tell the whole story. Many insects change their behavior based on chemical signals even though they have few olfactory receptors. Dogs were reported to have 1,094 olfactory receptor genes of which 20.3% are pseudogenes which leaves 872 unique, functional olfactory receptor genes (2). Pigs have 1,113 functional olfactory receptor genes and 188 pseudogenes (3) While it is tantalizing that dogs and pigs have a relatively large number of olfactory receptors, this alone does not mean that these types of species are any more or less driven by chemical signals than other species (insects being the best example of a highly-olfactory species with few olfactory receptors). Insects have on the order of 50–100 olfactory receptor genes while mammals have 300 to over 1,000 functional olfactory receptor genes (4).

We chose to compare dog and pig semiochemical biology because both species were domesticated many millennia ago and they are both known for their olfactory acuity. The objective of this interpretive review is to summarize and interpret much of the key scientific literature (but not the lay literature) on semiochemicals in pigs and dogs. We wish to provide the reader with background definitions and concepts that support current thinking about semiochemicals. Space limitations prevents an exhaustive review of all scientific and non-scientific published reports about semiochemicals in dogs and pigs. Before we review dog and pig semiochemical known biology, we felt it critical to give background information to define terms, report mammalian olfactory organs (which vary in anatomy and function among species), discuss what evidence is required to determine molecule(s) is/are a pheromone and we give one example from mice of a well-defined pheromone. This background information may help the reader interpret the dog and pig scientific literature.

## Definitions, overlap in terminology and biological examples

Chemical signals refer to a broad category of molecules or collections of molecules that change the physiology and/or behavior of an animal [and interestingly of plants, too (5)]. The term Pheromone was the first well-recognized term applied to this sort of communication. The term Pheromone was coined by insect biologists Karlson and Luscher in 1959 (6). They referred to pheromones as molecules, like hormones that communicate between an endocrine gland and distant tissues, communicating between two animals through space. They supported the definition that stands today, that pheromones are molecule(s) that send a chemical signal from one animal to another that changes the physiology or behavior of an animal of the same species. Since the early reports, we now know that animals have many ways of chemical communication beyond the definition of pheromone. Let's briefly review the terms in this field because we find many chemical signal molecules that are not pheromones (by definition), but they do send a message from one animal to another.

Semiochemicals is the broadest technical term (along with chemical signals) that can apply to any chemical communication between animals including insects and vertebrates (7) either within or among species (8). “Semio-” means signal; therefore, any chemical signal could qualify (9). Even the smell of food that attracts animals to eat is a semiochemical, but clearly not a pheromone. When a dog identifies the odor of another dog, that tells it this was left by an adult male dog (for example). In this example this chemical signal is not a pheromone, but an important signal. Within the basket of semiochemicals, we have pheromones, allomones, kairomones, attractants, repellants and interomones. The below definitions are from the scientific literature and Wikipedia except for the term Interomone which is a recently proposed term. The term Interomone defines a type of semiochemical that does not clearly fall within any of the other definitions (details below).

Briefly, an allomone (10) refers to a chemical signal that is released by one animal of a given species that impacts another species that benefits the originator but not the receiver. An example is the smell of a skunk. The skunk emits a chemical signal that detracts a potential predator which benefits the skunk but not the predator.

A kairomone (10) is a semiochemical that benefits the sender and harms the emitter. Cats find mice because mice emit kairomones that allow the cat to locate the mice. Some plants, for example, emit chemical signals that repel insects.

Attractants and repellants are important signals among insects. Many insect species have both alarm pheromones (11) and aggregation pheromones (12). We presume mammals have them too. One mammalian alarm pheromone has been identified (see below). Semiochemicals such as attractants and repellants are important for establishing normal behavior in that they move animals toward or away from food and/or danger, but they are not pheromones by definition.

In this interpretive review, we first searched databases for any paper on the broad topic of semiochemicals in dogs and pigs. We then categorized each paper as being a controlled scientific study (which were included) or not (ex., surveys or non-controlled studies). If past papers were reviewed in earlier published scientific reviews, we minimized discussion of these papers as they were adequately reviewed recently. Finally, we attempted to not just report scientific papers, but to interpret them in the face of the anatomy, physiology of each species and how they impact general semiochemical concepts. Before reviewing publications about dog and pig olfaction, some background information on olfactory organs and some cautions about types of evidence that are used to support calling a given molecule a pheromone (or other semiochemical) is given.

## Organs that sense semiochemicals

Confusion abounds in the reporting of semiochemical mechanisms and application methods. Semiochemicals are marketed for pigs and dogs including sprays, collars, room diffusers and ointments (among others). If the chemical signals work *via* the VNO, then the animal must touch a liquid and draw it in the nares or oral duct of the naso-palatine duct that leads to the VNO. Table 1 summarizes the likely olfactory mechanisms for different semiochemical delivery systems. If one knew the part of the olfactory system that is activated for a given semiochemical, then the most appropriate delivery form would be clearer.

**Table 1:** Physical form of semiochemicals that may activate three major olfactory sensory organs.

**Form of semiochemical**	**Olfactory Activation (the**		
	**SO and GG have not been**		
	**described in these species)**		
	**MOE**	**Trigeminal nerve**	**VNO**
Liquid spray	Yes	Yes	Yes
Collar	Yes	Yes	No
Room diffuser	Yes	Yes	No

Semiochemicals mechanisms to activate the brain requires activation of at least one and possibly more than one olfactory sensory system. One cannot speculate with any degree of certainty that a room diffuser or collar can activate the VNO, for example.

This section will review what is broadly termed olfactory organs. Taste is also a chemical sense but will not be reviewed here. We hope in the future more attention will be paid to the sense of taste, which is understudied in farm and companion animals. The olfactory organs were reviewed by others in recent years and so here we provide a simplified overview. See other reviews that go into more detail on olfactory organs (13, 14).

Why is it important to know which olfactory sensory organ or organ senses a given semiochemical? The first reason is that as scientists, we seek to understand mechanisms of action of semiochemicals and pheromones. On a practical level, each chemical sensory organ is stimulated by different sorts of chemical signals (15). Some signals are highly volatile and are sensed by one (or more) organs while less volatile signals are sensed when in a liquid form (15). This allows less-volatile molecules to be perceived as a chemical signal. Therefore, knowing which sensory organs a given chemical signal binds tells us about the behavioral biology (do animals have to touch?) and about how best to deliver therapeutic semiochemicals (ex., in aerosol or liquid form). A summary of current knowledge about the delivery form of chemical signals and the most likely sensory organs with functional receptors is simplified in Table 1.

The most extensive olfactory structure is the main olfactory epithelium (MOE) (16). The MOE of dogs and pigs is the largest, most extensive olfactory organ (13). Sniffing has been extensively studied in the dog (17) but not the pig. The olfactory receptor genes' reach are extended because of the millions of olfactory sensory cells that extend from the olfactory bulb to the MOE.

The Vomeronasal Organ (VNO) is the other major olfactory organ that is often discussed and studied in relation to semiochemicals and pheromones. Some authors and the public have the idea that the VNO functions to bind pheromones unlike the other sensory organs. This is simply not always true. We have many examples where the VNO is not necessary for a pheromone effect in multiple species. Yes, the VNO senses many pheromones, but not all (details below).

The VNO is highly functional, minimally functional, or absent in different mammalian species. Humans, for example, have some VNO receptor genes, but the VNO is not functional. In addition, both evolutionary and artificial selection can change the VNO anatomy and receptors over time. The VNO has three classes or families of chemical receptors (18). These receptor families are labeled V1R, V2R and FPR (VNO 1 receptors, VNO 2 receptors, and formyl peptide receptors, respectively). The MOE (but not the VNO) also has olfactory receptors such as trace amine-associated receptors (TAAR) but the TARR have not been examined closely in the pig or dog.

Other chemical sensory organs are less studied in farm and companion animals. The Gruenberg Ganglion (GG) lies at the inside tip of the nares (19). It senses alarm pheromones in rodents (19). It is better activated by liquid, less volatile molecules; a bit like the VNO. If an animal wets its nose with urine, it would have the opportunity to activate the GG organ and the VNO. These two olfactory organs seem designed to sense less-volatile molecules found in liquid rather than aerosol form.

The fifth cranial nerve, the Trigeminal nerve has chemical sensory receptors (more below). The Trigeminal nerve is more likely to sense volatile molecules much like the MOE. The human trigeminal nerve, for example, senses Androstenone (20). Could the same be true for other species?

The Septal Organ (SO) of Mesera is found in the nasal passage and contains chemical sensory receptors. Little is known about this organ in domestic animals. It certainly warrants further study; however, dogs and pigs do not seem to have a SO (13).

An elegant comparative anatomical study was made by the Salazar laboratory about the olfactory organs of the dog and mouse (13). They could not find the GG or SO in the dog. They also confirmed that the VNO of the dog is missing one class of VNO receptors (VR2) which suggested to them that the VNO of the dog is undergoing involution (perhaps due to domestication) which might have also happened to the GG and SO in this species. We do not know yet if other domestic animal species have functional or involuted VNO, GG or SO structures. The Salazar laboratory has documented that both pigs and dogs have a VNO and MOE, but they were unable to identify GG or SO structures.

We must remember that animals can have non-olfactory mechanisms for pheromones to change animal behavior or physiology. Androstenone (a widely studied mammalian pheromone) was shown to actually enter the blood stream (the nares and olfactory mucosa are highly vascularized) and directly bind to the hypothalamus and other brain tissues (21). Knowing that pheromones can directly stimulate internal brain organs is very interesting. We have much to learn about chemical sensory organs and mechanisms through which semiochemicals cause changes in behavior and physiology.

The biological literature has historically promoted the idea that the MOE is for sensing environmental volatile molecules while the VNO functions to sense pheromones. We now know that this is not always true (examples given in this paper), but especially we understand the MOE can sense pheromones (22, 23). The VNO responds more rapidly to evolutionary and artificial selection than does the MOE (18). Dog and pig breeds and individuals also vary considerably in olfactory sensitivity (24, 25). This variation in olfactory sensitivity could be used in breeding programs to improve olfactory detection by dogs and this variation may explain variation in pig productivity. Much is to be learned and applied in this area.

Chemical signal molecules and their receptors have been conserved in species from insects to mammals. Early scientists who studied pheromones believed that they were species-specific more-so than today. It did not make biological sense that the elephant and lion could have the same sex pheromone molecule if they lived in the same ecosystem (5). This could cause havoc in the animal kingdom (if so, lions might try to breed elephants). This view had to be re-assessed when it was discovered that the male and female sex pheromones in elephants are the same molecule used by insects as an aggregation pheromone (5). This finding caused a re-think of chemical communication. First, species along the phylogenetic tree from insects to mammals share biochemical signals and their receptors. Second, if two species evolved use of a chemical signal, but they occurred at different times or in different locations, this would not induce biological confusion. Third, it makes sense that volatile molecules and their receptors are conserved among species and used for different biological functions in one species vs. another.

We assume that many biochemical pathways are similar or identical in most species. This is an assumption that allows biomedical research on a variety of species that leads to overall physiological or behavioral principles. If a dog, horse, pig, and human all make the volatile steroid androstenone, then they are likely to have receptors for that molecule in their olfactory systems. That is not to say that this pig pheromone molecule is a pheromone in a horse, dog or human as it has been suggested in the pig. However, we do know that many species can smell and react to androstenone. Androstenone is one of the boar sex pheromone molecules, but it also changes the behavior of dogs (26), horses (27) and possibly humans (20, 28). There was not a name for such a semiochemical; hence, the name interomone was coined. An Interomone is a pheromone in one species that has a defined sender, receiver, and chemical signal receptors in a given species, but that also has a different effect on physiology and/or behavior of animals of another species (definition by the authors). Because many species share the same olfactory receptors, finding a pheromone in one species makes it a candidate for an interomone function in other species.

One study (28) looked at the human brain activation of women who were exposed to the boar pheromone androstenone, or the rabbit pheromone (2-Methyl-2-Butenal 2M2B) or rose odor (as a control). Interestingly, each molecule activated the post-olfactory brain of women in different brain regions. Each odor caused a signature response in the brain (28). Clearly, many humans can smell androstenone (at high concentrations for some) and 2M2B, but the interesting part is that they have different effects on the brain—which is a response expected if these chemical signals serve different functions in different mammalian species behavior and physiology. Androstenone activated the insula, amygdala, and the frontal and temporal neocortex—all areas for olfactory processing. These areas are activated in emotional experiences such as fear and affection. Exposure to 2M2B activated the cerebellum, the somatosensory cortex, the mother cortex, and the prefrontal cortex (including Brodmann's areas 6, and 8). 2M2B brain activation is consistent with what has been reported for pleasant odors (29). Androstenone brain activation is consistent with entering an emotional state (but we don't know if this is positive or negative emotions ranging from love to fear).

Androstenone (more on this molecule below) will stop dogs from barking (26) or leash pulling (30). We do not know of an anti-barking pheromone in dogs, yet the phenomenon is real—the molecule changes dog behavior. Androstenone has not been shown to be a pheromone in dogs. Horses become calmer when androstenone is sensed (27), but androstenone has not been described as a pheromone in horses. The Interomone tag holds this molecule's behavioral effects until we understand how this molecule not just changes behavior or physiology, but how it operates in the behavioral biology of the animal. Interomones currently identified can have considerably more powerful effects on behavior or physiology than pheromones (see below).

## What evidence is required to conclude a molecule is a semiochemical or a pheromone?

The science of semiochemicals in domestic animals is in its early stages. For most candidate molecule(s), the mechanism of action is not known (31), but at the same time, we can observe behaviors and see benefits by using semiochemicals to improve the lives of animals and people. It is an odd point in time in that semiochemicals have biological and commercial applications before their mechanisms of action are understood. This is backwards to typical drug developmental programs where the mechanisms are first understood, then therapies are developed.

In microbiology, for a given microbe to be called the cause of a disease, certain facts must be established (it has to be isolated from a sick animal and be able to induce illness in another, etc.). To be identified as a pheromone molecule(s), we propose the following evidence be collected to confirm that the given molecule(s) is/are a pheromone. A given semiochemical that is not a pheromone may still be useful in behavioral therapies.

Wyatt (5) surmised that the original studies that demonstrated a pheromone in insects (6) used as evidence the same scientific method as Koch who sought the microbiological cause of infectious disease. Wyatt suggested that such evidence is required before a molecule(s) is called a pheromone. We agree that this approach is needed in the field of semiochemicals and in particular to describe any given molecule(s) as a pheromone, interomone or other class of semiochemical.

Under this definition of Pheromone:

A Pheromone(s) is/are substances which are excreted to the outside by an individual and received by a second individual of the same species, in which they release a specific reaction, for example a definite behavior or a developmental process (6, 32). We add the caveat that certain molecules may be similar between closely related species (for example, the donkey and horse that can interbreed). Evolutionary biology does not favor two species in the same ecosystem using the same pheromone for the same purpose. This would cause biological confusion.

To demonstrate that a given molecule(s) is/are a pheromone, we propose one would have to provide this evidence:

1. The molecule(s) is/are secreted by an animal of that species at an appropriate time (for example, a female in estrus, or a stressed animal sending an alarm signal)
  a By definition, just having the molecule(s) is not enough. If a chemical signal is to change behavior or physiology, it should not be present (or absent; some chemical signals might involve lowering concentrations) all the time. For example, if an alarm pheromone is produced during a state of heightened stress, then that makes sense in that the receiving animal senses the alarm state. If the same molecule is found at the same concentration in a non-stressed/non-alarmed animal, then the molecule(s) cannot be a pheromone. Likewise, if an estrus-signaling molecule is present all the time, it cannot be an estrus pheromone. And if a maternal pheromone is found at the same concentration in non-lactating females, it is probably not a maternal pheromone. Pageat and Gaultier (31) put forward this concept another way when they said that “The right pheromone has to be chosen and emitted at the right time and on the right place so as to obtain the expected results.”
  b That a given biological fluid contains substances that change the behavior and/or physiology of an animal can be demonstrated prior to the identification of the actual molecule(s). This evidence is enough to hypothesize that a pheromone exists, but not enough to claim a given molecule is the actual pheromone.

2. The candidate molecule is produced by and received/perceived by a member of the same species
  a The candidate pheromone molecule must be produced/secreted/excreted by an animal of the species
  b A specific chemical sensory organ must bind the molecule(s)
    i Chemical sensory systems are discussed elsewhere here. Because there are several chemical sensory systems, it is useful, but not required, to know which organ(s) receptors bind the candidate pheromone.
  c There would have to be a specific brain activation associated with the signal receipt
    i This evidence is not required but would be supportive of a candidate pheromone. We are far from understanding the post-olfactory bulb brain activation signatures for semiochemicals, although early work is promising that showed different brain activation for semiochemicals in humans (28).

3. The molecule(s) must change the behavior and/or the physiology of the receiving animal
  a This is a critical step in identification of a pheromone. The candidate pheromone molecule(s) must cause some change in the receiving animal. One would think that this step is obvious, but it is not always documented.
  b If a semiochemical has not been shown to be a pheromone, but still changes animal physiology and/or behavior, it still can have value for animals and their owners.

While not absolutely required on the above list, if a given candidate molecule is shown to bind a particular olfactory sensory cell (such as the VNO or GG or MOE or others), then the evidence for it as a pheromone (or semiochemical) is stronger; but this alone is not sufficient to claim it is a pheromone. Most people can smell lavender, but that does not mean it is a pheromone.

The literature does not contain definitive data showing many mammalian chemical signals are pheromones. Still, a given chemical signal can have important effects on animal behavior and/or physiology without the need for controlled studies to demonstrate it is in fact a pheromone. The problem today is that many people recognize and believe they understand pheromones and so when marketing a semiochemical the term pheromone may be used when in fact the behaviorally-active chemical signal was not demonstrated to be a pheromone through controlled studies. We discuss later the idea that if a semiochemical benefits animals, does it really matter what it is called (pheromone, interomone, semiochemical)?

Let us review a mammalian pheromone and the evidence that they might be or are a pheromone. This example case is for the mouse alarm pheromone.

## An example of a well-defined pheromone

The road to pheromone discovery often begins with assessment of biological fluids. In 1972 Rottman and Snowdown published studies where they reported that odors for stressed mice caused other mice to behave differently (33). Removal of the olfactory mucosa removed this effect. These findings provide evidence for criteria 1 above. The work did not identify a candidate or actual pheromone molecule; however, it provides good support to search for the responsible pheromone. Later, scientists working in Switzerland identified the mouse alarm pheromone in an elegant series of studies (34). These authors describe how the alarm pheromone bound to neurons in the Grueneberg ganglion (GG; a sensory structure near the tip of the mouse nares) which when activated causes changes in mouse behavior consistent with an alarm state. The GG has distinct chemical receptors and also shares some receptors with the VNO. The authors described how the alarm pheromone activated GG cells and caused both physiologic and behavioral changes. Interestingly, other pheromones and biological fluids did not activate the sensory cells of the GG. In the original paper published in 2008, they showed that the mouse alarm pheromone was sensed by the GG (a potential interomone effect; or a kairomone). A few years later in 2013, the same laboratory identified the mouse alarm pheromone (35) in a series of studies that showed that 2-sec-butyl-4,5-dihydrothiazole (BT) was the mouse alarm pheromone. They showed that BT changed mouse behavior after screening several unique molecules found in biological fluids of alarmed mice. If the only evidence required was that the molecules were present during an alarm state, then we would have about 8 molecules as the alarm pheromone. However, they showed in the 2013 paper that only 1 of the 8 molecules activates sensory neurons and changed behavior. Taken together, these studies support all three criteria (and other criteria such as showing that the candidate alarm pheromone binds the GG receptors) proposed above for concluding that the candidate molecule is an alarm pheromone. They also showed that alarm pheromones are sensed by other species and that alarm pheromones in relates species are in a similar class of water-soluble organic molecules that are not highly volatile and would best be perceived in liquid form (such as urine). Before we review pig and dog semiochemical science, some general principles should be examined so we can place the published evidence in a proper light.

### Cautions

While semiochemical scientific data are widely published, we have access to much more than scientific information about semiochemicals. Anecdotal, video, commentaries and print materials abound about pheromones and semiochemicals. The casual reader can be easily confused or convinced by inadequate evidence. In addition, the placebo effect with semiochemicals is very real and easy to demonstrate (Supplementary material S1). This means that selling water or a vehicle can be effective in changing animal behavior in many cases. If a report does not control for the placebo effect, and if the study is not double-blinded, then one cannot be sure that the effect is due to the active molecules (Supplementary material S1).

Many companies sell semiochemicals for pigs and dogs. Marketing can be effectively used to stimulate interest in semiochemicals even in the absence of scientific evidence that it is a pheromone or a semiochemical (Supplementary material S2).

A final caution is outlined in Supplementary materials against accepting certain anecdotal and even patent claims as scientific evidence. Patents are designed to protect an idea, not to demonstrate that the idea is efficacious in a population of animals (Supplementary material S3). In this review, we put the most weight on data from peer-reviewed, scientific papers.

## Semiochemicals in domestic pigs

### Introduction to pig semiochemicals

A recent review of pig pheromones was published in 2021 (36) which followed an earlier review (37). Here we attempt to review primary studies that are more recent or that take a different approach to a given pig pheromone or interomone. Here we also provide negative data from our laboratory that has not been published to clarify what is currently known. We encourage readers to review the earlier reviews to get an understanding of literature related to pig semiochemicals.

The domestic pig has an extensive MOE and a functional Trigeminal sensory neurons and a VNO, but the GG and SO have not been found in the domestic pig (14). The pig VNO has only V1R receptors, but it does not have V2R or FPR receptors (18, 24). The VNO is small in the pig, but functional at least for chemical signals that might bind V1R receptors. Authors have not reported in the pig (as they have in the dog) that the pig VNO is undergoing involution, however, its lack of 2 families of VNO receptors indicate it is not as robust as the VNO of other species such as rodents (38).

### Boar sexual pheromone

Adult boars emit a strong odor from their secretions, especially from their saliva (39). The smelly boar is sought-out by a sow in estrus. If the sow is not in estrus, the boar odor has little effect on the sow. Thus, the boar is a constant emitter of semiochemicals while the sow responds to these odors only when she is in heat (14).

The domestic pig was the first species for which a mammalian pheromone was proposed. In the 1960s, Patterson, a food scientist, reported that androstenone was responsible for the off-smelling odor from the meat of male pigs. In 1971, Melrose et al. suggested that androstenone stimulated sows in estrus to show sexual behavior (40). Androstenone is one molecule produced by adult male pigs in large quantities. Again, not all the criteria were met to establish in 1971 that androstenone is the boar pheromone. Other molecules are also found in boar saliva. In the 1970's, a commercial product was produced containing androstenone to cause sow sexual behavior. Farmers thought it was useful, but not as good as a boar. Something seemed wrong. A few years later, other steroids were shown to be present in boar saliva. 5α-androstenone and 3α- and 3β-androstenol were also shown to all be present in boar saliva (and a few more steroid variants) (41) and to induce sexual behavior in sows in estrus (40). Even with the application of the two most effective molecules (androstenone and androstenol), sow sexual behavior was not enough to be as good as a live boar. Thus, our laboratory re-examined this issue. We hypothesized that with this pheromone being a mixture of molecules, that one or more molecules might be missing from the reported pheromones.

When we examined saliva from both boars and sows, we identified three molecules that were unique to the boar and not found in sow saliva. In addition to androstenone and androstenol, we identified a non-steroid molecule in boar saliva—quinoline. We then determined the effects of each molecule individually and in combination in a behavioral study of nearly 1,000 sows (42). Each of the three molecules when sprayed on each sow's snout induced different behaviors in the sexual behavioral repertoire. In this blinded, randomized trial, the greatest sexual behavioral response of sows in estrus was observed when all three molecules were included. We then showed in a large-scale field test that these three molecules can induce sexual behavior and improve reproductive rates of weaned sows (43). All three pieces of evidence are published to demonstrate that these three molecules comprise the boar sexual pheromone that induces a behavioral response in sows in estrus. This story is not complete, however, because when we use the novel pheromone containing all three behavior changing molecules, the sow response is still not quite as good as a live boar (42, 44); all three molecules do induce a richer set of sexual behaviors than any single molecule. Although many sows respond to the boar pheromone with a full sexual behavioral response, boar stimuli including the sight and sound and perhaps touch of a boar seem necessary for the complete sexual response in some sows. Quinoline was not found in the saliva of boars from some farms (44). We have since learned that quinoline is highly volatile and in transport from a boar site to our laboratory resulted in loss of quinoline; one can argue that quinoline has a more powerful effect on the sexual behaviors of estrus sows than does androstenone or androstenol (43). The loss of active molecules is a problem with field samples of volatile semiochemicals. In all boars tested [6 total (44)], where we quickly cap the saliva and assay it in a timely manner, we have always found quinoline. Secondly, we showed that quinoline alone causes some sow sexual behaviors when they are in estrus and when combined with androstenone and androstenol give as stronger sow sexual behavioral response than the steroids alone (42).

We do have additional information on at least one molecule in the complete boar pheromone. Much work has been done on androstenone in the pig and in other species. One question is: which olfactory organ(s) bind the boar sex pheromones? While one might expect the VNO to be the primary site of androstenone binding causing behavioral changes, this is not the case. Dorries et al. in 1995 showed that female adult pigs could sense androstenone at a 5-fold lower concentration than males (45). The same research group then showed that androstenone effects on sexual behavior were not mediated through the VNO (or at least the VNO is not required), but rather they suggested the MOE is the site of androstenone binding (46) and sow sexual behavior initiation (and that the VNO is not needed for androstenone effects). They did not examine the possibility of androstenone activating the trigeminal nerve sensory cells.

The androstenone story gets more interesting. Some humans can smell androstenone and some cannot. Researchers have reported that androstenone binds to the human trigeminal nerve (the 5th cranial nerve) (20). We do not know if the pig also perceives androstenone *via* the trigeminal nerve. Earlier work (46) reported androstenone works *via* activation of the MOE and not the VNO; however, that work did not differentiate potential binding of androstenone to the trigeminal nerve sensory cells or directly in the brain. The pig has specific olfactory binding proteins (OBP) in its nasal mucosa and MOE and VNO. Nagnan-Le Meillour et al. reported that these OBPs specific for binding androstenone and that these extracellular binding proteins are conserved in species from insects to other mammals (47). This means that many species can sense androstenone and this molecule can have behavioral effects that may be surprising (at this time)—a view consistent with the Interomone concept. We know that pigs, humans, horses, dogs, and cats all change behavior when presented with androstenone (48).

After mentioning in an earlier patent [discussed in Supplementary material S3, the lowest form of proof of a concept; (49)] from this laboratory, androstenone was observed to change horse and dog behavior. Choi et al. reported that androstenone induces horses to be more compliant in human-horse interactions (a calming effect) (27). The same authors earlier showed that androstenone can change the brain chemistry of horses by changing blood serotonin, cortisol, and β-endorphin concentrations (50). Androstenone also stops dogs from barking or jumping or leash pulling (26, 30). We do not know if androstenone is a pheromone in dogs, horses, or humans, but investigators have reported changes in behavior and physiology after androstenone exposure in each of these species (26, 27, 48). This molecule clearly modulates different behaviors in different species. Because many species have the ability to produce and sense androstenone and related molecules, it is a good candidate for evolutionary uses for different purposes in different species (51).

### Maternal-neonatal pheromone in the pig

A pig pheromone review written by Sankarganesh et al., in 2021 did not focus on, but mentioned, maternal-neonatal semiochemicals (36). Here we provide additional information.

A product (Suilence) was sold as a pig maternal pheromone (called pig appeasing pheromone; PAP) a few decades ago. PAP molecules have OBPs in the MOE and the VNO (52). This means that pigs can sense the molecules; but without other controls, this is not sufficient evidence to claim it is a pheromone (we can smell a rose). Authors generally agree that OBPs are more tuned to pheromones than to other more general odors (53).

When applied to weaned pigs, PAP reduced aggression and increased feeding behavior in a controlled setting (54, 55). Given its lack of commercial success, one must assume it did not perform as well on commercial farms. However, PAP origins and development provided interesting data that add to our understanding of maternal-neonatal pheromones (MP). The inventors of PAP did not show that these fatty acids increased during lactation. In fact, in our published studies, we found all the PAP molecules (except Myristic Acid) in skin secretions and feces of pregnant sows. This suggested to us that PAP may not be the functional maternal-neonatal pheromone (but we cannot say this is the last word on this subject as the science continues).

Julie Morrow-Tesch was a PhD student at Texas Tech University and sought to use step 1 in our “evidence for a pheromone” above (56, 57). She examined which biological fluids/substances were more attractive to piglets and when they learn their mother's smell. She found that piglets learn their mother's odor by about 12 h old (they could not recognize their mother's odor at birth). She also found that lactating sow mammary skin secretions, feces and urine were preferred by piglets compared with secretions from pregnant sows. In addition, when she washed the semiochemicals off the sow's udder, piglets could not or would not nurse. These findings confirmed to us that there truly is a maternal-neonatal pheromone in the pig. Additionally, these findings are consistent with the idea that piglets require a MP from birth, they cannot nurse without them (56), but they then learn their mother's odor signature by 12 h of life (this odor signature is technically not a pheromone).

Sow feces was highly preferred by piglets over other biological fluids. Patrick Pageat (PP) in concert with others, collected skin secretions from lactating sows and showed the presence of several fatty acids (58). We are not aware of any actual published scientific studies showing these molecules alone or in combination are MPs (based on the criteria above). Still, we applied this putative MP to weaned pigs and had a positive effect on feeding behaviors and a reduction in fighting (54). However, when this product was tried on commercial farms, it had minimal or no efficacy for the weaned pig (we believed the commercial environment was too odorous and thus required a stronger olfactory signal). We then examined the rabbit MP (2-Methyl-2-Butenal, 2M2B) as a potential Interomone and, again, found a reliable reduction in fighting and an increase in feeding (59) in our controlled university high-health farm. Again, as with PAP when we took 2M2B it to the field, we could not replicate its positive effects with a large number of commercial pigs. Both positive studies (PAP and 2M2B) were performed in a high-health, clean, university environment. Commercial farms have many more pigs in a room and the weaned pig rooms are often more odorous than the university rooms. The concept seemed good, but at that point, we did not have a viable semiochemical candidate powerful enough to overcome the issues in a commercial environment. So, we started over.

We considered our steps/studies 1–3 above. These critical studies had not been performed. We did not know that the PAP pheromone was produced only during lactation. We did not know the concentrations in biological fluids and substances. As a precursor study (as in #1 above), we removed the feces from sows from piglet birth and for 1 week after. We found that piglets deprived from maternal feces grew slower and had more health problems than control litters that had access to sow feces (60). Of course, many other explanations could be proposed other than the loss of MPs (such as a microbiome effect). But it gave us enough evidence to move forward—piglet health and growth is better if sow feces are present.

We then sought to determine the unique molecules found in sow secretions/excretions, that were found only during lactation. The first issue is that pregnant sows are limit fed while lactating sows are often full fed and the diets are different (all of these factors can impact semiochemicals). We fed sows the same quantity and quality of feed from late gestation through lactation. Then, we collected fluids and feces from pregnant and lactating sows. Morrow-Tesch et al., had shown piglets prefer feces from any lactating sow over feces from pregnant sows (56, 57). After extensive chemistry, Aviles-Rosa et al. showed that the *lactating sow* has only 2 unique molecules in her feces—Skatole and Myristic Acid (61). The fatty acids in the PP patents attributed to the lactating sows were also found in pregnant sows, so these are less likely to be the MPs.

Next, we used a weaned pig model to show that weaned pigs exposed to the sow MP, fought less, ate more, and grew a bit faster (61). We now know that a range of concentrations and application methods of the sow MP can improve pig performance and welfare. Recently, scientists from Iowa State gave peri-farrowing sows our MP and they found an improvement in pre-weaning survival of 5% (this is a large economic value). The idea now is that if we can increase the MP concentrations, piglets may improve their maternal bond and when used after weaning can ease the stress of weaning.

Currently, our thinking is that the skatole attracts piglets toward an area of higher concentration, that is, they chemotax toward skatole (such as feces and udder secretions). Myristic acid (which is far less volatile than skatole) is then sensed when they get closer to the source, much like a piglet moving toward an udder and teat.

### Other pig semiochemicals

Many more pig semiochemicals and pheromone remain to be discovered. Decades ago, biological fluids from pigs were shown to modulate aggressive and submissive behavior in pigs (62, 63), but follow-up work has not been performed to identify the specific molecules. Semiochemicals that would be of value to the pig and pig producers include appetite stimulants, alarm pheromones, aggressive and submissive behavior modulation, sow-in-estrus pheromones, semiochemicals indicative of illness, and aggregation and dispersal semiochemicals (which would be useful in handling pigs). Some work has been done on these possible semiochemicals, but not enough to warrant inclusion in this review. Semiochemicals that indicate either general sickness or that specific disease would be useful as a non-invasive diagnostic tool especially as a part of newer smart barn technologies.

We have some indications that pig semiochemicals can be measured for the detection of disease. We know that sometimes we can smell that they are sick. Each infectious and metabolic disease should cause a certain semiochemical signature that might include volatile molecules that could be detected remotely (ex., by biosensors, or by doing the chemistry). To get an idea if this is possible, Devaraj from our laboratory organized a study where we gave pigs a fever by injection of LPS, then we determined if we could find “fever” molecules. Several candidates were identified (64). Our hypothesis is that each disease state should produce unique volatile molecules that can be sensed. Imagine if the air leaving a pig barn could be sampled to immediately know if the pigs have a certain disease. Keep in mind that this idea is not dependent on having DNA or RNA from an infected pig. Smart barns of the future are expected to have such technologies.

## Semiochemicals in dogs

### Introduction to dog semiochemicals

The dog is an interesting species in that it was the first animals to be domesticated. Dogs are known for their keen sense of smell even though breeds of dogs are known to vary in olfactory acuity. Dogs with large, elongated noses have large nasal cavities to house the olfactory epithelium which dictates the number of olfactory receptor cells (OR) available to the dog (65). Certain breeds of dogs have high or low numbers of ORs because of the anatomical structure of their skull and nose (66). The shape of the nose influences the surface area of the olfactory epithelium and the number of ORs the dog is capable of housing. Breeds like the Bloodhound have very large ORCs (300 million), while brachycephalic breeds have poor olfactory dexterity (65, 67). The area of the olfactory epithelium ranges from 18 to 150 cm^2^ (68, 69). This superior range can be compared to a human's, at 3 cm^2^, to show how enhanced the dog's olfactory senses are (70).

The dog has been subjected to artificial human selection for millennia and during that time evolutionary pressures have changed the dog olfactory system compared to other canids. Olfactory acuity or sensitivity may be influenced by the genetic differences and variability of olfactory propensity due to a transition in breeding for working (hunting, herding, guarding) to breeding for appearances (25, 67). Several authors agree that the dog's vomeronasal organ has undergone *involution* over time. The VNO has three major classes of receptors. The dog has a (surprisingly) small number of V1R receptors and is devoid of V2R VNO receptors (71). The dog may have 1 or 2 FPR VNO receptors (18), but this needs to be confirmed. Some genes naturally mutate over time. If the mutations favor survival, they remain in the genome. If a gene mutation benefits survival, then it will be retained in the genome. If the gene mutation inactivates its function, and there is no disadvantage to the animal, then the mutated gene, called a pseudogene, remains in the genome. V2R receptors, in the dog, are now pseudogenes. In contrast, another canid, the fox, has retained V2R receptors (72).

The domestic dog has an extensive MOE and a functional Trigeminal sensory neuron and a VNO, but the GG and SO have not been found. The VNO is small in the dog, but functional at least for chemical signals that might bind some V1R receptors. People have documented in the dog that the VNO is undergoing involution; however, its lack of VNO receptors indicate it is not as robust as the VNO of other species such as rodents. Horowitz et al., determined that the dog has a reduction in detection/attention to odors because of the natural inter-specific relationship with humans. Their current domestic environment does not reward for olfactory recognitions; instead, dogs have begun to use behavioral cues (73).

The understanding of the capabilities of the dog's olfactory sensory has come a long way since the 1950s. Adrian in 1948 speculated that there was little difference between the olfactory acuity between the dog and humans (74). Today, dogs utilize their olfaction senses for military, health, conservation, and more than 30 different drug detection tasks (25). Though some breeds of dogs are more equipped for scent detection, no specific breed has been bred solely for detection (67). Due to the high variability in breeds, methods of detection, and influence of detection by personality traits and perceptual learning abilities, the depth of olfaction potential in the dog is not completely understood (66). Amyl Acetate has been tested to find the threshold of detection in dogs in a researched controlled setting. The findings ranged from 40 parts per billion (ppm) to 1.5 parts be trillion (75). Aliphatic acids have been found to have a large threshold between 10,000 and 100,000 ppm (76). The olfactory sensitivity or lower limit of detection is different for each chemical signal for which the animal has olfactory receptors.

The Salazar laboratory published an elegant study comparing the olfactory organs in the dog and the mouse. They did not find the GG or SO organs in the dog as they did in the mouse (13). The VNO of the domestic dog was believed to be undergoing involution while wild canids retain a more functional VNO. The dog's primary olfactory sensory organ is the MOE, not the VNO. The VNO may still function in the dog, but certainly not like it does in other VNO-dependent species.

In much of the literature, the VNO is described as an organ that senses pheromones. This is clearly not always the case (see Androstenone effects on sensory organs above). In the case of the dog, some dogs are bred or kept as sniffing dogs that are expected to have heightened olfactory acuity. This can be the case as long as the odors are aerosolized and able to be sensed by the MOE and not the VNO, the GG, or SO of the dog. To be sure, one would have to do the anatomical and molecular biology work as the Salazar laboratory did for different breeds and genotypes/phenotypes of dogs.

Any person interested in semiochemicals in dogs and cats should carefully read the review by Pageat (PP) and Gaultier (31). This review contains information that summarizes the history of pheromones in mammals and published work up to that point in time, but it also gives much background information on chemical sensory organs and the development of pheromones that are now sold commercially. The authors introduce the word Pheromonotherapy which describes the use of pheromones (we would say semiochemicals) to treat behavioral disorders. Clearly, the PP semiochemicals have helped animals and are a commercial success.

This early review (31) includes statements and ideas that we now believe may not be true. For example, this early review suggests that the VNO is the main site of perception of pheromones; we know that this is not always the case in the pig and the dog (see above text). The concept of involution of the dog VNO was not yet proposed when this review was published. However, this review provides a rich and informative overview of the olfactory systems in the dog and cat. The description of the VNO anatomy and how it functions is elegantly written.

Over a decade ago, Frank et al. published a systematic review of dog and cat appeasing pheromones (77). Among qualified scientific studies they reviewed, 12 out of 14 were negative or neutral toward companion animal pheromones. They found that most studies did not support the use of DAP to reduce anxiety during noise, travel, or veterinary visits. This was surprising because by that time, DAP was widely purchased around the world as a therapy for various behavioral problems. Because they summarized literature prior to 2010, we will focus on more recent literature which overall is more positive toward DAP. Many dozens of papers have been published in DAP; too many to review here. We will not review published surveys and consumer marketing studies that lack scientific rigor. We found no mechanistic papers that may determine if DAP is actually a pheromone. The field of Pheromonotherapy (it may be awkward to say Semiotherapy) in dogs suffers due to people who either strongly believe or do not believe that they work. Scientific proof must rise above the placebo effect.

Pheromonotherapy has been suggested for use in dogs with various behavioral problems and in various situations. Model systems have been developed to study separation anxiety, car transport, veterinary visits, and loud noises (ex., thunderstorms) among others. If you take any one of these models, it is clear that not all dogs experience stress from the same situation. Not all dogs have thunderstorm phobia. Thus, when one selects a population of dogs, but does not pre-screen them for thunderstorm phobia, then one would not expect all dogs to respond to any therapy—especially if they do not have the problem to start with. This adds to the lack of clarity in the data. Not all dogs are stressed in a shelter, during a vet visit, or during travel. Then, even if we started with a population of dogs known to have thunderstorm phobia, one would not expect all dogs to respond the same because they would have different levels of the condition. When a study reports “no effect” we have to ask: Did they start with dogs with the diagnosed condition? Did all dogs have the same degree of stress? Also, was the study placebo controlled and blinded and did it have sufficient power to detect biological differences?

### Dog sex pheromone

Reproductive behavior in dogs (and other species) was reviewed by Root Kustritz (78). In the late 1970s, Methyl p-hydroxybenzoate (HB) was reported as a sex pheromone in female dogs released when they are in estrus (79). This molecule was reported to be sold to people wanting to assist dog breeding. A few years later, another laboratory reported 2 unidentified molecules (not HB) were associated with the bitch in estrus (80). Surprisingly few studies have been performed to clarify semiochemicals associated with reproduction in the dog.

### Dog appeasing “pheromone”

The dog appeasing pheromone (DAP) is a collection of fatty acids reported and patented by PP starting over 2 decades ago (31). Many patents have been issued with the same general theme; fatty acids found on or near mammalian mammary glands were identified and when applied to dogs (or other species) they propose to decrease stress, anxiety, and aggressiveness in mammals, among others (81). The ratio and composition changes with species. Here we focus on the dog appeasing “pheromones.”

The DAP has been the subject of many publications, both clinical/scientific and surveys (discussed below). DAP also enjoys great market success and has a good image among some scientists, veterinarians, and consumers. However, we could find no scientific evidence that actually shows that DAP is a pheromone in the dog; it certainly is a semiochemical. Yes, mammary glands and surrounding tissues produce fatty acids. Do they preferentially produce them in lactating mammals and not non-lactating animals? What does one do with the large number of studies reporting no effect of DAP? Among neonates, does DAP change the behavior or physiology of suckling animals or their mother? These are largely unanswered questions. We do know that DAP molecules have OBPs in the MOE and the VNO (52). The fact that dogs can perceive a given odor is not sufficient proof that it is a pheromone (we can smell bacon cooking, but this is not a pheromone). However, it is positive that dogs can clearly sense DAP molecules in some situations and that they can have (often positive) effects on dog behavior.

Some studies included objective measures of physiology in their assessment of DAP. In one case, Australian scientists determined if during dog separation from its owners (a stress), heart rate variability was influenced by therapeutic interventions. They found that music and lavender improved heart rate variability, but DAP had no effect (82). They did report some behavioral improvements with the use of DAP in this stress model. The same group found improvements in stress-related behaviors with the use of DAP sprayed on a cloth collar for dogs in a shelter environment (83). In another examination of physiological effects of separation from its owner from the UK, the elevation in heart rate or behavioral signs of distress associated with owner separation was not changed by a DAP diffuser (84).

Shelter dogs often experience stress. Tod et al. applied DAP or a placebo to shelter dogs (85). They found a significant reduction in barking with DAP exposure. This effect was small compared with the effects of the interomone that stops dogs from barking (26).

Sometimes studies use a collar while others use a spray or a room diffuser. The dose would be quite different in each application. Also, with a collar or room diffuser, the VNO is unlikely to be activated as it requires a liquid or droplets to activate the VNO. These studies suggest that DAP works through the MOE or the Trigeminal nerve. This would have to be tested in future studies. To consumers, the fact that it works is more important than which olfactory organ is required to be activated.

One cannot ignore the many studies about DAP efficacy that were positive or negative especially in the last decade (too many to review here). We would classify DAP as a semiochemical at this stage and not a pheromone.

### Interomones in dogs

The interomone concept is described above. What follows here could be called an Interomone, but these molecules are clearly semiochemicals. Once volatile molecules were observed as shared between insects and mammals (5), and also understanding that most species can smell semiochemicals from other species, it makes evolutionary sense that many mammals share chemical signals and receptors for those signals. That does not mean that they are pheromones. But they can have powerful effects on behavior and physiology.

The pig sex pheromone stops many dogs from barking, jumping, leash pulling, and perhaps other behaviors one wishes to modify or stop (3, 6). This does not mean that androstenone is a pheromone in the dog. As an Interomone, androstenone can have a much more rapid and powerful effect on dog behavior than any putative pheromone we have tested. DAP was shown to have a small effect on reduction of barking in dogs as well (85); but a transient effect on the reduction in barking is not as significant as the effects of Androstenone that immediately stops dogs from barking (26). Given in a pulsatile manner, Androstenone can be used to train dogs to not bark (or to not do certain behaviors).

The second Interomone used in dogs (and cats) is 2M2B—the rabbit maternal pheromone. Clearly dogs, cats, and most humans can smell 2M2B. It is found in many foods and plants and most animals. It is highly volatile, and it changes dog behavior and physiology in meaningful ways. Some literature is still being generated, but here we provide an update.

Pirner completed her PhD at Texas Tech University in 2018 where she studied dog pheromones and interomones. She recorded both behavior and heart rate (by telemetry). She exposed dogs to a control or 2M2B in two models: car transport or a simulated thunderstorm. These are highly controlled studies done in a laboratory setting in rooms designed for semiochemical research (fresh air in only; no air exchange between rooms) (28).

**Figure 1:**
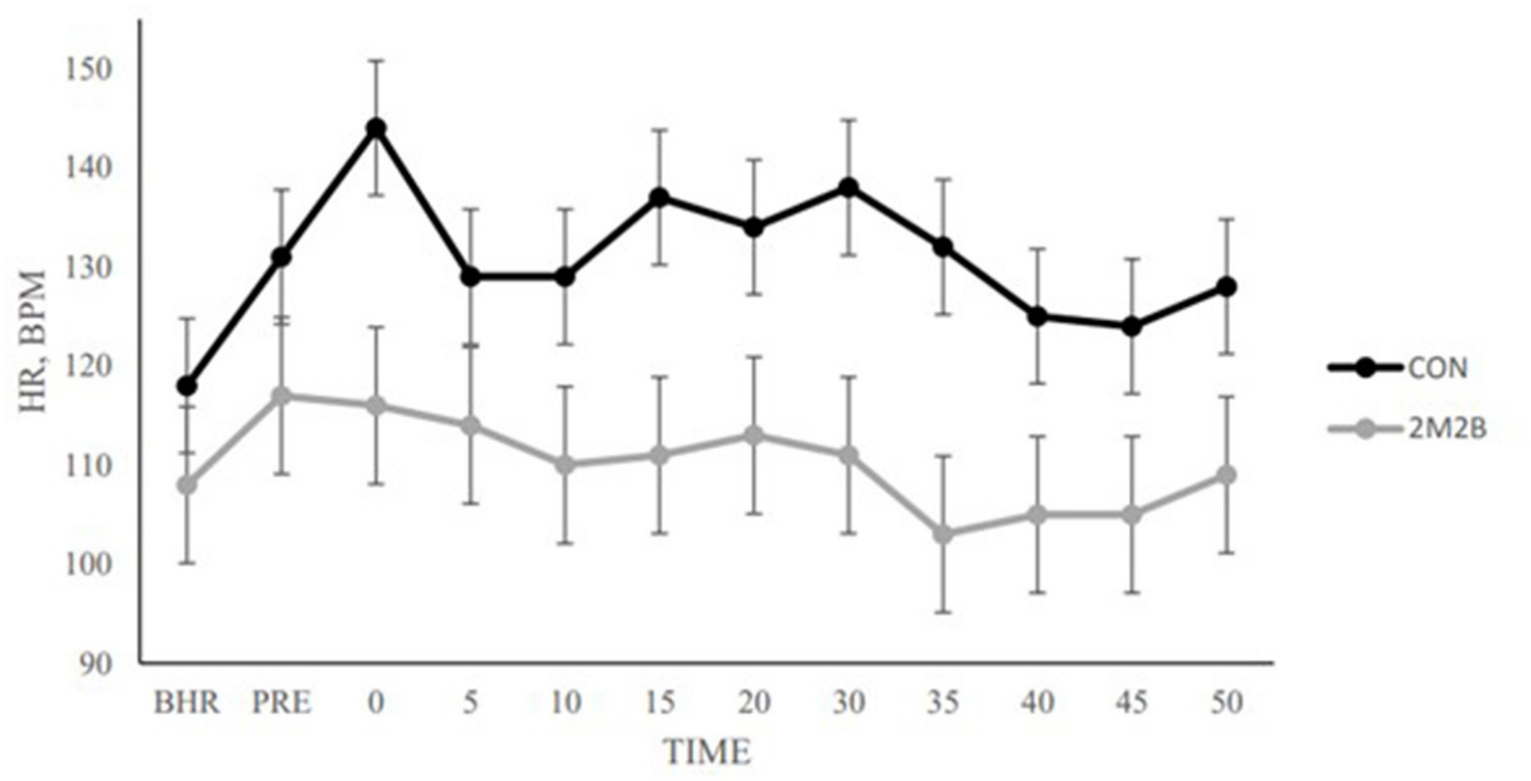
Dog heart rate when dogs either had a control car trip or a car trip in which 2M2B was sprayed in the environment. Each data point represents 8 dogs. Note that dogs with 2M2B therapy had significantly lower heart rate than control dogs. Adapted from Pirner (28). BHR is basal heart rate. Pre is immediately before transport. Note that control dogs increase in HR at time zero and remain elevated during the trip. This is an objective demonstration of a calming effect.

In the car transport model, dogs typically increase their heart rate which could indicate excitement or stress. During a 50-min car ride, dogs with 2M2B spray had significantly lower heart rate than during the control trip (Figure 1). Along with a powerful effect on preventing the rise in dog HR, transport changed dog behavior and 2M2B provided some intervention (28). With 2M2B on board, transported dogs had a lower HR and spent less time lip-licking and vocalizing (whining).

**Figure 2:**
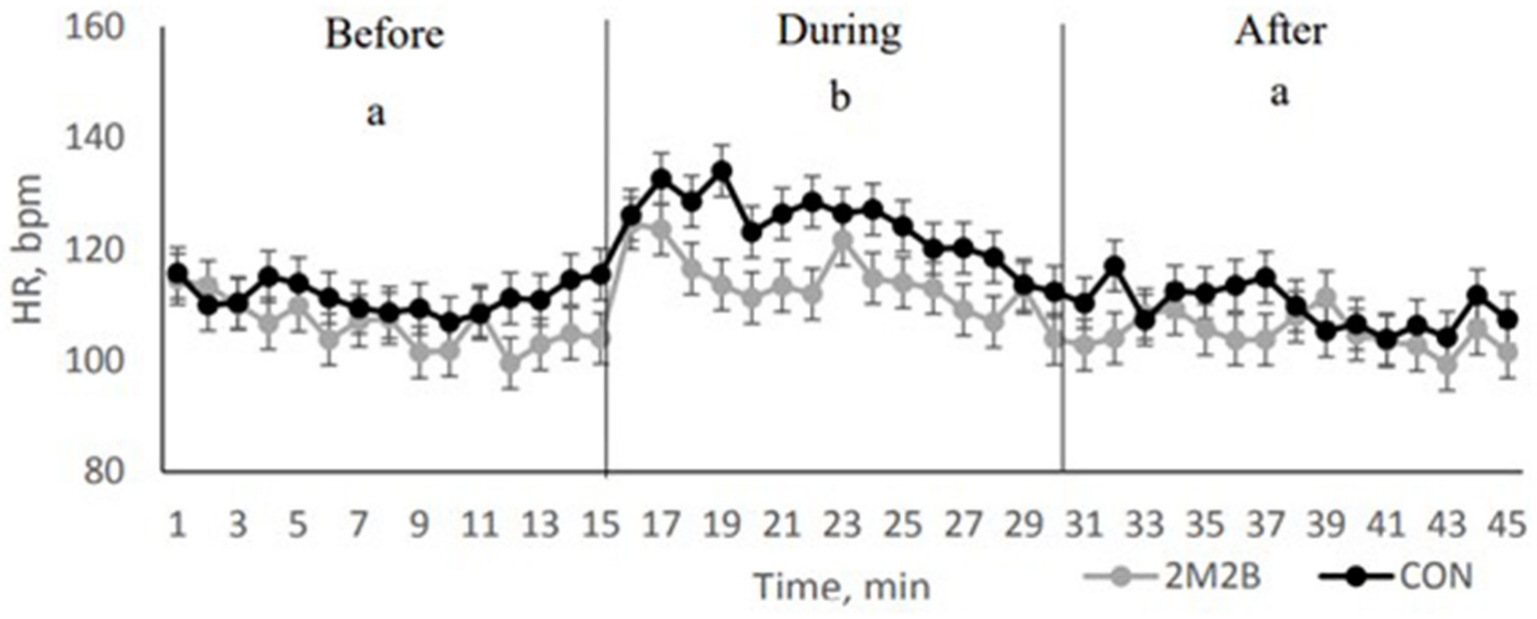
Dog heart rate when dogs either had a control or 2M2B treatment before during and after a simulated thunderstorm. Each data point represents 19 dogs. Note that dogs with 2M2B therapy had significantly lower heart rate than control dogs (or the rise in HR was blunted). Adapted from Pirner (28). Before is immediately before the loud thunderstorm. Note that control dogs increase in HR at time zero and remain elevated during the trip. This is an objective demonstration of a calming effect.

In the thunderstorm model, the same general findings were generated. Dogs were exposed to a recording of an actual thunderstorm that was played at over 100 dB. Each dog had a control or a 2M2B experience. The rise in HR associated with the thunderstorm was blunted when 2M2B was on board (Figure 2). Dogs with 2M2B spent more time laying down during the stressful thunderstorm. With the objective measures of heart rate and time spent lying down, once could say that 2M2B calms dogs.

Scientists have studied dog HR and HR variability in dogs when DAP was used (82). Dog behavior, HR and HRV were not significantly changed by use of DAP in a shelter environment. A research group replicated that DAP did not change HR in a separation anxiety model (84). Note the powerful effect of the interomone (discussed above) on HR in models of dog stress in contrast to a lack of major effect on HR with DAP.

### Other semiochemicals in dogs

We expect that dogs have many semiochemicals that they use in their natural behavioral biology. Dogs clearly scent mark and overmark urinary signals. These may relay information like age, sex, general health, and when the animal last was at that place. Using these to direct dog urination would be useful. For example, if an effective semiochemical would be on a paper pee pad, it could direct dogs to urinate in that place. This could be useful especially when the dog is in an unfamiliar environment.

Sex pheromones are surely found in dogs. Because these have limited commercial value, work in this area has not continued.

Dogs may have alarm pheromones. These would have commercial success if discovered. For example, if dogs chewed on furniture, an alarm pheromone might deter this behavior.

## Comparison of pig and dog olfaction and future research

Another measure of chemical sensory function is the relative olfactory acuity or sensitivity of pigs and dogs—two species known for their keen sense of smell. Olfactory sensitivity often means the ability to detect low concentrations of chemical signals. The pig can detect n-butanol (a component of manure) at a 2,000-times lower concentration than humans; pigs could detect this molecule at an average of 20 parts per trillion (86). In our work with boar sex pheromones, we found some pigs could detect these pheromones in behavioral tests down to < 8 parts per billion (51), with significant individual variation in olfactory acuity. Olfactory acuity depends on the actual chemical signal (87). Some species have more olfactory receptors and are expected to be able to sense more molecules. Beyond the numbers of chemical receptors, receptors evolve and either increase sensitivity or become pseudogenes over time and due to evolutionary and artificial selection pressures. Pigs and dogs are known to vary among breeds in olfactory acuity (24, 25). Pigs have one of the largest numbers of functional olfactory receptors [over 1,100; (3)] which suggests that the olfactory genome is stable or expanding in the domestic pig while the dog has fewer olfactory receptor genes (88).

Dog olfaction has been studied for over 100 years. Even early-on, both the MOE and the Trigeminal nerve sensory cells were known and used for examination of olfactory mechanisms (89). Few current studies consider the dog Trigeminal nerve sensory functions in studies of dog olfaction. Authors in the field of behavioral therapy seem to focus on the dog VNO which is an organ that is in the process of involuting. Dog olfactory research is active as dogs are used to detect a range of semiochemicals such as drugs, bombs, cadavers, disease states, infections and more [ex., (90)]. It would behoove workers to increase attention to the dog MOE and the Trigeminal nerve activation rather than the VNO for most useful purposes. Dog sniffing which is the behavior they show when detecting volatile molecules would likely bind the MOE and/or the Trigeminal nerve, but not the VNO.

Comparing pig and dog olfactory sensitivity is challenging. Even if one species has a more sensitive olfactory system for one molecule, that could not be the case for a different chemical signal; however, both species can detect olfactory molecules lower than the parts per billion levels (87). One cannot say universally that either the dog or pig have greater olfactory acuity, nor is this an easy question to answer.

Pheromone products that hope to improve pig and dog welfare are often delivered as a spray, room diffuser, or collar. These methods of delivery would not likely bind the few VNO receptors that both the dog and pig have. The VNO requires a liquid to be drawn into the organ for it to be useful (as in Flehmen). This cannot easily happen with an aerosol. One would have to spray directly in the mouth or nares or allow the animal to lick or rub on the product if the intention is to activate the VNO.

In many regards, the pig and the olfactory systems are similar. Key similarities include, first, an emphasis on semiochemicals activating the MOE and the Trigeminal nerve (and in some cases directly binding the hypothalamus), but less-so the VNO and certainly not the GG or SO because they have not been identified in pigs or dogs. Second, both species have a large number of olfactory receptors (although pigs have more than dogs). Third, both species can detect molecules at part per trillion or lower concentrations. Finally, both species use semiochemicals and their olfactory systems as a major part of their sensing of the world. Clearly, semiochemical research in both pigs and dogs is just starting. Bringing both science and reason to the field will accelerate interesting new discoveries that can improve animal health and welfare.

We suggest a few areas that are fertile for new discoveries about semiochemicals. First, the anatomy and function of the olfactory organs would be useful to know when thinking about how to deliver semiochemicals (spray, liquid, etc.). Second, we have virtually no information on the brain processing of olfactory signals within and beyond the olfactory bulbs. Chemical sensory processing post-olfactory bulb is not the same for each chemical signal (28). Third, new semiochemicals and pheromones are ripe for discovery in dogs and pigs (and other species). This review may help scientists map out a plan for discovery of new chemical signals. Finally, behavioral problems are significant in domestic animals. Finding semiochemicals that can assist with breeding, modulate aggression and other behaviors, and reduce stress could result in non-drug interventions. Providing olfactory enrichment should be a goal for those that wish to improve the sensory environment of domestic animals, especially pigs and dogs.

In the evaluation of animal welfare, semiochemicals should be a major part of that evaluation. Some semiochemicals may stress or induce fear in animals. Others may reduce stress and make the animals “feel” more calm or comfortable. Clearly, animal welfare assessments do not emphasize the olfactory environment when they evaluate facilities, people, and animals. Yet, both pigs and dogs are strongly influenced by semiochemicals. We suggest that the olfactory environment be considered along with the physical environment when animal welfare is assessed. And like any other animal therapy, the welfare effects (positive or negative) of application of semiochemicals should be assessed to be sure we are creating an environment that is consistent with the biology of domestic animals.

## Author contributions

JM conceived the idea, organized the sections, interpreted the data and was responsible for the major thoughts in this interpretive review. CA and MH conducted literature reviews and annotated bibliographies and wrote portions of the paper. All authors approved the final version of this paper.

## Conflict of interest

Author JM received grant support to conduct research in this field. He is the inventor on patents related to pheromones. Some patents generate royalty income in the field of semiochemicals. The remaining authors declare that the research was conducted in the absence of any commercial or financial relationships that could be construed as a potential conflict of interest.

## Publisher's note

All claims expressed in this article are solely those of the authors and do not necessarily represent those of their affiliated organizations, or those of the publisher, the editors and the reviewers. Any product that may be evaluated in this article, or claim that may be made by its manufacturer, is not guaranteed or endorsed by the publisher.
