# Supplementary material for: Interpretive review: Semiochemicals in domestic pigs and dogs
Source: Front Vet Sci. 2022 Oct 25;9:967980. doi: 10.3389/fvets.2022.967980 (PMC9640746; doi:10.3389/fvets.2022.967980)
Supplement: Supplementary file 1 [file Data_Sheet_1.PDF]

frontiers

Supplementary Material

1 Supplementary Data

S1: The placebo effect is real in semiochemical research

Animal behavior is more variable than many performance measures like growth and reproductive rates. The coefficient of variation (CV) for measures of weight gain is 30% or less; the CV for behavioral measures is often over 50% (for example, in (1) the CV for ADG was 18%; while in (2) the CV for agonistic behavior was 109%). The response to putative semiochemicals can be highly variable as well. One can easily demonstrate the placebo effect in studies attempting to demonstrate efficacy of a semiochemical or pheromone. Especially for companion animals, people have a desire for behavioral therapies to be beneficial and this will unknowingly bias even reasonable people.

An example of the placebo effect is seen in a paper showing the interomone androstenone will stop dogs from barking (3). Two types of placebo effects were demonstrated in that paper. Certain kenneled dogs were shown to bark and jump when a human and a dog stood in front of the kennel. The human-teaser dog pair then left and returned. When nothing was done, 25% of the dogs did not bark/jump upon the second visit. When the spray can contained only the vehicle and not the androstenone, 33% of the dogs stopped barking. This is clearly the placebo effect. When the same spray was applied to barking/jumping dogs, 100% of the dogs stopped barking/jumping. The placebo effect was one-third the effectiveness of the active interomone.

Now imagine giving a consumer a can of a semiochemical to use and report on its efficacy. If the study does not include appropriate treatments to control for the placebo effect, then one would expect a meaningful percentage of dog owners will report it is efficacious due to the placebo effect alone. Part of the placebo effect in home-self-reporting studies is the desire for people with animals with behavioral problems to want the treatment to work (4). In some cases, the animal responds to a change in its environment – like a water spray in their direction. Due to the high variation in behavioral measures, it is difficult to measure responses accurately and reliably. Also, behavioral therapies treat individuals and individuals are expected to vary in response to semiochemicals just as not all humans respond to drugs that work on most people and some drugs work on just a few people in the population. While semiochemicals are not drugs, they should be subjected to the same requirement to demonstrate efficacy and safety (5, 6). Efficacy data are available for many pig and dog semiochemicals. While we have not found safety studies published, the fact that millions of pigs and dogs have been treated with semiochemicals and no ill effects have been reported tells us that these products are safe.

S2: Commentary: Marketing Semiochemicals

The applications of semiochemicals to change/improve animal behavior are important to improve animal health and welfare. The technologies are considered clean, green, and ethical (7). Semiochemicals are considered less onerous than drugs to some consumers, so they use them to see if this technology can reduce the need for drugs or extensive training. Unfortunately, many products are sold to consumers without stating the active pheromone and without providing any sort of proof that it may perform as intended. One test of any putative pheromone is to see of the science supports

its application. At another level, the market reception tells us something about consumer acceptance. Pheromones are not government regulated (as are drugs) in most countries and so anyone can market anything without efficacy or safety data. One could market water and would get the placebo effect. This means that water would get sales and even repeat customers even if the product is not effective beyond its placebo effect. The plethora of poorly-defined products with limited scientific data confuses retailers and consumers. The marketplace for companion animal semiochemicals is confusing to everyone. Some honest, logic and scientific standards should be applied to help farmers, retailers and consumers understand semiochemicals. When marketing gets ahead of the science, animals may benefit while scientists may be frustrated with the lack of understanding the underpinnings of semiochemical mechanisms.

Beyond the placebo effect, one would not expect any given semiochemical or pheromone to work in all animals. First, one must be sure the animal has the behavioral problem that is trying to be treated. A dog treated for anxiety that actually does not have anxiety would not be expected to respond as an anxious dog might. If you spray an effective pheromone, it should not work in all animals. Animals vary in the degree to which they can perceive odors, they vary in how extreme the behavioral problem is, and the application method may not be appropriate or effective. Most drugs are not effective in all people with the given condition. So, we should not expect semiochemical therapies to be effective in all animals. Catnip does not change all cats' behavior. Drugs to treat depression are not effective in all dogs. Semiochemicals should not be expected to work in all animals; however, we do expect that science can show that they are effective in a high percentage of animals.

When a company sells a product without knowing that it is effective as advertised, some people will still buy it, perhaps because of the placebo effect. Some consumers will believe it works. Animals can be helped with behavioral intervention and training, and if a placebo helps solve the problem, then these animals would be helped. While it is scientifically unacceptable, animals can benefit from marginally effective or ineffective therapies due to slick marketing and the placebo effect. Corporations, however, are at risk of legal action if products are sold that have not been shown to live up to the claims on the label.

One other caveat of semiochemical therapies have to do with the claims made on the label. For companion animals, many semiochemicals are sold as "calming" products. The word calming actually has no legal or regulatory definition (in the USA), so it is difficult to show that any product calms an animal. We should be able to show that a product changes behavior or physiology in an objective study. If the regulatory burden applied to drugs was applied to semiochemicals (which would not happen in the USA with our current laws), the cost to market such products would be much higher.

S3: Levels of proof

In new discoveries, the world has several levels of proof before a semiochemical or pheromone can be considered to be real and biologically or medicinally relevant. Here we discuss the levels of proof afforded by different supporting types of evidence so the reader can navigate information.

The standard of proof to obtain a patent is one of the lowest levels of proof that is required. Replication is positive in a patent application, but not required; one must simply "reduce the idea to practice" – which could be one example. In some cases, an idea can be patented with minimal data. Patents also include much more range of dose and methods with no data to support the range of

effective dose and the specific methodology. A study may report that a pheromone concentration of 1% is effective, so the patent author will state, and the patent examiner will accept, a range of effective concentration from 0.001 to 10%, for example. This added vagary protects the inventor from people simply making a minor change in dose (for example) and obtain a new patent. Being vague also is a way to keep a trade secret. We would consider the patent the lowest level of proof of any concept.

Another level of proof is afforded by surveys of users of the products. One can ask people who use the product to rate its efficacy. This evidence is not scientific evidence, but such information should not be dismissed out of hand. Some scientific concepts do not translate into real-world efficacy. Many examples abound of something working in the lab and not in the field. When consumers report that a given product is efficacious, it gives some indication that the product works in the field (the home or farm, for example), but this is not scientific proof of efficacy – it is evidence that marketing was effective. One could market a placebo and even get repeat customers because the placebo effect is real. Many products are on the market for which little, no or negative scientific evidence of efficacy can be found. We have an ethical issue here in that some people may believe that it is not right to market products for which no scientific data supports its efficacy. Others can argue that if a placebo works in 1 out of 3 animals, this is still a benefit that is derived from minimal animal intervention (no spray or drug or semiochemical). One can argue that the least intrusive therapy should be applied. If the placebo is enough to gain efficacy, then this is the least intrusive therapy. The problem comes when someone markets product making claims that are not supported with data. Semiochemicals are usually not regulated, so they can be used with no evidence of efficacy and safety. Because pheromones are not regulated (in the USA by the FDA), anyone can market anything that they claim has some health or behavioral effect if it is not regulated. The only recourse for such products is truth-in-selling statutes regulated by federal governments. However, few products are ever subjected to challenge based on truth in advertising laws. Because of the issues with consumer survey data, these will be reviewed only briefly as such studies do not provide scientific evidence of efficacy.

The next level of proof is found in studies that report field data with appropriate control groups. Randomized, double-blind field studies can yield valuable data about efficacy and safety of products. One cannot rely on consumer impressions because consumers are not trained to collect objective measures. This issue may be resolved if the investigators enter the homes or field to collect uniform data. These studies are very expensive and time consuming to do correctly. They are often not done well due to the large cost of such studies.

The next and highest level of evidence is to have the work published in scientific journals that use peer reviewers and have editorial control over contents. Most scientists believe this is the only evidence that is acceptable. We must keep in mind that some journals are not reputable, and some are considered predatory journals. Not only do we seek peer-reviewed scientific journal articles, but the journal must pass muster as being up to international standards for science.

To be marketed as an effective product that is safe, objective, published, and scientific data are needed for each product. This includes demonstration that it is a semiochemical (based on science) and that it works in the real world.

We must remind the reader that; many semiochemical products labeled as pheromones and sold for use in domestic animals can have significant benefit to the animals even though these molecules have not been determined to be a pheromone.

2 References for Supplementary Materials

1. McGlone, J. J., G. Thompson, and S. Devaraj. 2017. A natural interomone 2-methyl-2-butenal stimulates feed intake and weight gain in weaned pigs. Animal. Published online 13 July 2016. In print February 2017 issue. 11:306-308 doi: https://doi.org/10.1017/S1751731116001154

2. McGlone, J. J. and D. L. Anderson. 2002. Synthetic maternal pheromone stimulates feeding behavior and weight gain in weaned pigs. J. Animal Science. 80:3179-3183.

3. McGlone JJ, Thompson WG, Guay KA. CASE STUDY: The pig pheromone androstenone, acting as an interomone, stops dogs from barking. The Professional Animal Scientist (2014) 30:105–108. doi: 10.15232/S1080-7446(15)30091-7

4. McMillan, F. D. 1999. The placebo effect in animals. JAVMA 215:992-999.

5. McGlone JJ, Thompson WG, Guay KA. CASE STUDY: The pig pheromone androstenone, acting as an interomone, stops dogs from barking. *The Professional Animal Scientist* (2014) 30:105–108. doi: 10.15232/S1080-7446(15)30091-7

6. Wyatt TD. Fifty years of pheromones. Nature (2009) 457:262–263. doi: 10.1038/457262a

7. Martin G. The ‘Clean, Green and Ethical’ Concept in Animal Production. Agrociencia Uruguay [Internet]. 2009 Dec 10 [cited 2022 Jul 23];13(3). Available from: http://164.73.52.167/ojs/index.php/agrociencia/article/view/840
